# Spatial-explicit modeling of social vulnerability to malaria in East Africa

**DOI:** 10.1186/1476-072X-13-29

**Published:** 2014-08-15

**Authors:** Stefan Kienberger, Michael Hagenlocher

**Affiliations:** 1 Interfaculty Department of Geoinformatics – Z_GIS, University of Salzburg, Schillerstraße 30, Salzburg 5020, Austria

**Keywords:** Malaria, Vulnerability, Climate change adaptation, Integrated spatial modeling, Geon concept, Regionalization, Eastern Africa

## Abstract

**Background:**

Despite efforts in eradication and control, malaria remains a global challenge, particularly affecting vulnerable groups. Despite the recession in malaria cases, previously malaria free areas are increasingly confronted with epidemics as a result of changing environmental and socioeconomic conditions. Next to modeling transmission intensities and probabilities, integrated spatial methods targeting the complex interplay of factors that contribute to social vulnerability are required to effectively reduce malaria burden. We propose an integrative method for mapping relative levels of social vulnerability in a spatially explicit manner to support the identification of intervention measures.

**Methods:**

Based on a literature review, a holistic risk and vulnerability framework has been developed to guide the assessment of social vulnerability to water-related vector-borne diseases (VBDs) in the context of changing environmental and societal conditions. Building on the framework, this paper applies spatially explicit modeling for delineating homogeneous regions of social vulnerability to malaria in eastern Africa, while taking into account expert knowledge for weighting the single vulnerability indicators. To assess the influence of the selected indicators on the final index a local sensitivity analysis is carried out.

**Results:**

Results indicate that high levels of malaria vulnerability are concentrated in the highlands, where immunity within the population is currently low. Additionally, regions with a lack of access to education and health services aggravate vulnerability. Lower values can be found in regions with relatively low poverty, low population pressure, low conflict density and reduced contributions from the biological susceptibility domain. Overall, the factors characterizing vulnerability vary spatially in the region. The vulnerability index reveals a high level of robustness in regard to the final choice of input datasets, with the exception of the immunity indicator which has a marked impact on the composite vulnerability index.

**Conclusions:**

We introduce a conceptual framework for modeling risk and vulnerability to VBDs. Drawing on the framework we modeled social vulnerability to malaria in the context of global change using a spatially explicit approach. The results provide decision makers with place-specific options for targeting interventions that aim at reducing the burden of the disease amongst the different vulnerable population groups.

## Background

Mosquito-borne infectious diseases, such as malaria or dengue fever, impose a heavy burden on human health, and vulnerable populations in particular. In spite of the tremendous progress that has been made in reducing malaria endemicity over the past decade [1,2] there were still an estimated 207 million cases and approximately 627,000 malaria-related deaths in 2012 [2]. According to recent estimates by the World Health Organization (WHO) approximately half of the world’s population was at risk of malaria in 2012, with the countries of sub-Saharan Africa facing the highest risk [2]. In line with the global recession in malaria cases and deaths, malaria incidences have reduced over much of East Africa [3], but have resurged in eastern African highland locations with increased variability in disease rates [4-10], increasingly affecting areas with significant population numbers and densities. The causes of the resurge are controversially discussed in literature. Several papers have been published attributing this resurge to changes in environmental and climatic conditions in general [7] and climate variability in particular [9,10]. For example, it is widely accepted that increasing temperatures have direct impacts on both life-cycle stages of the *Anopheles* vector and the *Plasmodium* parasite [11]. Although subject to large model uncertainties [12], the projected changes in regional climate conditions [13] and the resulting increase in temperature and precipitation above the minimum temperature and precipitation thresholds of malaria transmission [14] might thus result in further spread and distribution of the disease [15,16].

Other studies, however, suggest that these effects do not act in isolation, and that other non-climatic factors, such as increase in resistance of the malaria parasite to drugs, or the decrease in control activities are more likely to be the driving forces behind the malaria resurge in this region [1,5,6]. Evidence has shown that the socioeconomic status (e.g., age, poverty, education, etc.) and development status are also fundamental determinants of malaria risk [17-19]. Huldén et al. [20], for example, point out that malaria being a tropical disease is a common misperception. They highlight that, although these are areas where the disease remains prevalent, it used to occur throughout all climate zones. According to their findings temperature has only a minor impact on malaria prevalence, while they found social factors, such as household size to be more important. This is also underpinned by Carter and Mendis [21], who declare that the malaria recession in Europe and North America in the 20th century is primarily attributable to a decline in human-vector contact as a result of changing living conditions and rising prosperity as well as changes in land use.

Independent of the controversial debate whether highland populations are immunologically at particular risk [22] or not [23], it is essential for the planning of targeted interventions to have up-to-date information on both (i) the spatial distribution of the disease and current endemicity levels, and (ii) the prevailing social vulnerabilities of the population. Thus, next to environmental (including climatic) factors that influence the spatial distribution of malaria, it is important to also take into consideration the range of socioeconomic, demographic, political, and behavioral factors that impact people’s susceptibility and (lack of) resilience to the disease [17-19]. Several papers have been published on factors that influence the spread and spatial distribution of the disease [21,24], including eastern Africa [25], and there are a few papers assessing malaria risk, that, besides environmental factors, also integrate socioeconomic and demographic factors [9,26-29]. To date, however, only few studies have been published on vulnerability to vector-borne diseases [17,18,30-32], and malaria in particular [9]. Wandiga et al. [9] carried out surveys in three communities in the Lake Victoria Basin (eastern Africa) to assess the role of climate change and its variability, hydrology and socioeconomic factors for malaria vulnerability on a local level. A spatially explicit approach for modeling, exploring and visualizing homogeneous units of social malaria vulnerability on a policy level for districts, countries or regions is, to the best of our knowledge, not existent yet.

This paper presents a conceptual and methodological framework for modeling social vulnerability to malaria in a spatially explicit manner for the regional scale. Based on a holistic conceptual risk and vulnerability framework that was developed to guide risk and vulnerability assessments for water-related vector-borne diseases, and a set of malaria-specific spatial indicators and indicator weights, we delineate homogeneous regions of social vulnerability to malaria for the eastern African region. The aim of the proposed approach is to provide information for the place-specific targeting and prioritization of interventions.

## Materials and methods

### Study area

**Figure 1:**
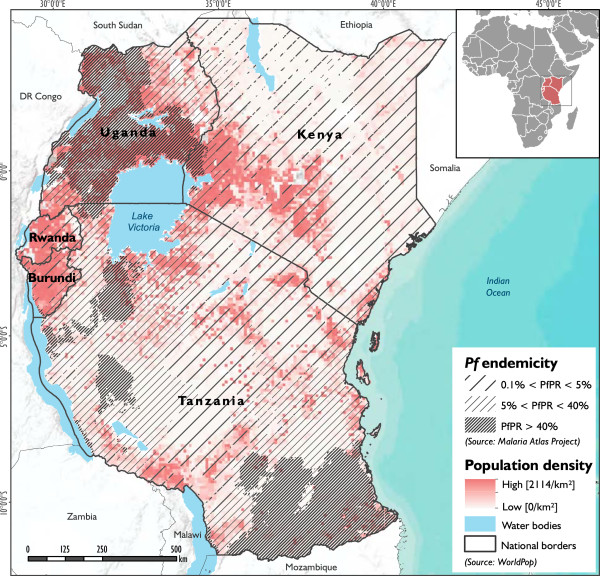
**Location of the study area.** The map shows the population density in the study area (in shades of red), overlaid with *Plasmodium falciparum* (*Pf*) endemicity levels (Gething et al. [24]) grouped in three different categories (as indicated by the solid lines).

The study area comprises the five countries that form the East African Community (EAC), i.e., the Republic of Kenya, the Republic of Uganda, the Republic of Rwanda, the Republic of Burundi and the United Republic of Tanzania. It covers an area of approximately 1,817.7 thousand square kilometers, including water bodies. As shown in Figure 1, the size and population density of the five countries varies tremendously. Rwanda (26,300 km^2^) and Burundi (27,800 km^2^) are the smallest countries, while Tanzania (939,300 km^2^) is by far the largest country, accounting for more than 50% of the total area of the entire EAC region [33]. As well as the country size itself, the average size of sub-national administrative units also varies significantly across the countries, which causes difficulties when comparing these units spatially. According to recent population projections, the five EAC countries have an estimated population of 134.5 million inhabitants [33], and account at certain locations (Rwanda, Burundi, Uganda) for one of the most densely populated regions on the continent [7]. This holds particularly true for the areas surrounding Lake Victoria and the southwestern part of Kenya, which are also areas of high malaria endemicity (see Figure 1). As a result of its relatively high overall population growth rate of 2.6% and enduring conflicts in the region, the population of the area is expected to further increase in the coming decades, thus forcing more people to resettle into areas that favor malaria transmission [32]. The region faces great spatial and temporal variability in terms of climate [7,10] and eco-regions. As a result of the generally high altitude in these regions, temperatures are relatively modest compared to other equatorial regions, with lower temperatures in the highlands (maxima of around 25 °C, and minima of 15°C at an altitude of 1,500 m) and higher temperatures in the humid coastal areas. As a result of global and regional climate change, the entire region has been confronted with rising temperatures and increased frequency and magnitude of extreme weather events [13]. In combination with increasing resistance of the malaria parasite to drugs, and a decrease in funding for vector control, this has resulted in a spread of malaria into areas that had not previously been exposed to the disease [4,5,10,34]. Figure 1 shows the spatial distribution of *Plasmodium falciparum* (*Pf*) malaria stratified by endemicity class for 2010 [24]. It highlights that malaria has already expanded into the highland areas, presenting epidemics beyond the lowland limits where the mosquito vectors are usually found [5-10].

### Framing risk and vulnerability to water-related VBDs

The concept of risk and specifically vulnerability is promising for linking malaria prevention and response with development agendas, as it helps to identify potential intervention options for reducing overall risk and strengthening resilience to VBDs independent of current disease prevalence. It provides valuable and necessary information for the malaria prevention and control community which often has to rely solely on information on current transmission or endemicity levels based on environmental factors, thus pursuing a reactive approach to reducing the malaria burden.

Concepts and terminologies of risk, vulnerability and related terms such as resilience or adaptive capacity are manifold and vary between different schools of thought. Within the climate change research arena, the previous IPCC (Intergovernmental Panel on Climate Change) approach [35,36] conceptualized vulnerability as a function of exposure, sensitivity, and adaptive capacity [37]. Contrarily, the disaster risk reduction (DRR) community defined risk as an integrative concept defined by vulnerability, exposure and hazard. Studies in the context of public health either use (the previous) IPCC-based concepts [9,32,38-40], or understand risk simply as the likelihood of disease occurrence [41,42].

With the latest IPCC assessment reports [43,44] a significant change in the understanding of risk and vulnerability in the context of climate change adaptation has been achieved. They stress that risk management, adaptation and action on climate change should be placed in the context of a planning and analysis framework that considers societal issues along with environmental factors. Understanding disease risk management as a social process allows for a shift in focus from responding to disease prevalence alone, towards an understanding of disease risk. This requires knowledge about how human interactions with the natural environment lead to the spread and prevalence of diseases, and how society is vulnerable to the potential burden of these diseases. Such an approach requires an understanding of the vulnerability of the population, including the allocation and distribution of social and economic resources that can work for, or against, the achievement of reduced diseases impacts [43].

Against this background we developed a holistic conceptual risk and vulnerability framework which (i) considers the notion of multiple inter-related factors contributing to disease risk, (ii) provides a clear framing of risk and vulnerability in-line with current IPCC recommendations, (iii) establishes a clear link to risk governance, climate change adaptation and related intervention measures, (iv) allows the identification of possible development pathways, and finally, (v) provides a holistic view of disease risk considering spatial and temporal scales.

In the framework (Figure 2.1), risk is defined as the potential occurrence of harmful consequences or losses (i.e., the potential burden of diseases) resulting from interactions between VBDs and vulnerable conditions of differential population groups. In line with the MOVE framework [45], the proposed framework reflects the multi-faceted nature of vulnerability, accounting for key causal factors such as susceptibility and lack of resilience.

**Figure 2:**
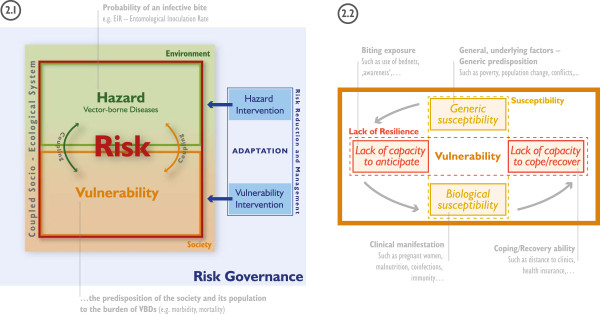
**Conceptual risk and vulnerability framework.** Risk framework and its integration within risk governance, climate change adaptation and associated intervention measures (2.1) and domains of social vulnerability (2.2) with illustrative examples.

A ‘hazard’ in the context of water-related VBDs is defined as the potentiality of disease occurrence which may have a negative impact on social assets in a given area and over a given period of time. Hazards include latent conditions that can represent future threats and are characterized by their location, magnitude, frequency and probability. An example for malaria is the probability of an infective bite, which can be represented through the Entomological Inoculation Rate (EIR).

Vulnerability is defined as the predisposition of the society and its population to the burden of water-related VBDs, considering spatial and temporal differences in susceptibility and lack of resilience [30,46]. Vulnerability largely rests within the conditions and dynamics of the coupled socio-ecological system exposed to VBDs. However, due to its multi-faceted nature it is mainly linked to societal conditions and processes. In our framework, vulnerability is seen as a dynamic process which represents the conditions set by the environment and the characteristics and actions of the vulnerable populations themselves. Dynamic is understood as the change of factors of vulnerability (and risk) over time.

The framework (Figure 2) was designed to be holistic in a sense that it can be applied to guide the assessment of risk and vulnerability to several water-related vector-borne diseases, such as malaria, dengue fever, schistosomiasis, Rift Valley fever, etc. at different spatial or temporal scales. Depending on the disease that is addressed, different indicators (and indicator weights) for modeling disease risk and/or vulnerability might be relevant. Here, the framework was used to guide the assessment of vulnerability to malaria on a regional scale. In this framework, vulnerability rests largely within the social dimension, which, to our understanding, encompasses various socioeconomic and demographic factors, and could be extended to institutional, ecological or cultural dimensions; and vulnerability is defined by susceptibility and lack of resilience. Susceptibility represents the propensity of societies or humans to be negatively affected by a VBD. Thereby we distinguish between generic susceptibility (SUS) and biological susceptibility (BIO). Generic susceptibility encompasses general underlying factors and the general predisposition of societies to malaria (e.g. poverty, population change, conflicts, etc.). Biological susceptibility relates to the clinical manifestation of malaria, which depends for instance on malnutrition, disease co-infection and/or immunity [30].

**Figure 3:**
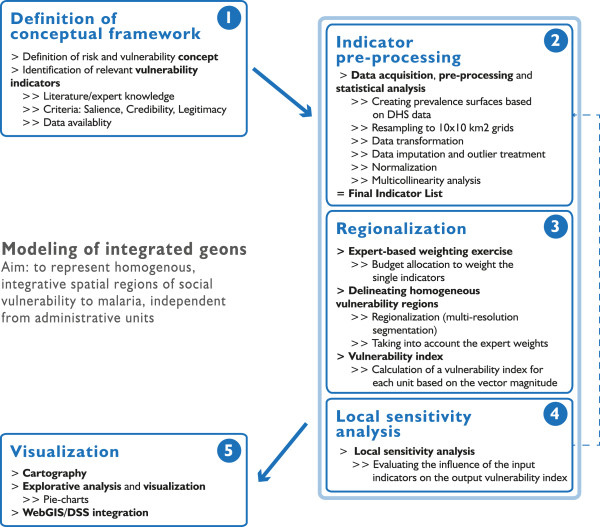
**Modeling workflow.** The workflow shows the individual modeling stages from the conceptualization to the visualization of vulnerability to malaria.

Lack of resilience refers to the lacking capacity of societies and population groups to respond and absorb negative impacts as a result of the lacking capacity to anticipate, respond to and recover from diseases [30]. Compared to adaptation processes and adaptive capacities, these capacities focus mainly on the ability to maintain the system’s functionality in light of VBDs impacting the society or system [45]. Adaptation (see Figure 2.1) deals with the ability of a community or a system to learn from present and past disease outbreaks and to change existing practices for potential future changes in environmental and societal conditions. Anticipation (C2A) itself entails a coherent set of strategies or programs and social capital available before the disease hazard arises and deals mainly with the reduction of biting exposure (e.g. use of bed nets, awareness, early warning systems etc.). Coping (C2C) refers to the ability of people, organizations, systems and/or communities to use available skills and resources to face and manage adverse conditions arising from endemic and epidemic diseases (such as distance to clinics). Whereas, recovery (C2R) refers to the capacity to restore adequate and sustainable living conditions, as well as having the capacities to overcome or manage the disease in a way that allows living in a physically healthy way (e.g. the availability of adequate treatment and health insurance).While the proposed framework can be adapted to various disease contexts, we assume a step-wise dependence of the different domains of social vulnerability to malaria, as indicated by the grey arrows in Figure 2.2. This is also reflected in the workflow outlined in Figure 3.

Through the frameworks’ integrative, while at the same time decomposable nature, it serves as a ‘guidance tool’ for the identification and development of systems of indicators of risk and vulnerability relevant for assessments at different spatial and temporal scales. Depending on the VBD that is addressed, a different set of indicators and indicator weights could be used to assess risk and vulnerability to the disease.

Additionally it helps to identify targeted intervention measures – at the hazard and vulnerability level – with the ultimate aim to reduce risk to VBDs.

### Vulnerability indicators and related datasets

Based on the outcomes of a systematic review of literature, the consultation of several domain experts at a series of expert consultations, and data availability, a preliminary set of 15 vulnerability indicators representing the social dimension of vulnerability to malaria was identified to reflect present day conditions (Table 1). All of them vary spatially in the study area.

**Table 1:** **List of vulnerability indicators**^a^

**Indicator name**	**Date**	**Resolution**^**b**^	**Sign**^**c**^	**Weight**	**Data source**
*Generic susceptibility (SUS)*				*0.2744*	
Number of women	2010	1 km	+	0.0272	AfriPop:demography
Population change	1970-2010	2.5-arc minutes	+	0.0314	GPWv3, UNEP-APD
Travel time to closest urban center	2000	30 arc-seconds	+	0.0229	JRC/WorldBank
Distance to roads	2010	Line layer	+	0.0286	OSM, ESA GlobCover, SRTMv4
Conflict density (km^2^)	1997-2009	Point layer	+	0.0429	ACLED
Number of people living on less than 2 USD per day	2010	2.5 arc-minutes	+	0.1214	CGIAR CSI
*Capacity to anticipate (C2A)*				*0.2671*	
Secondary/higher education (%)	2007/08	Point layer	-	0.0571	DHS
Child did not sleep under net last night (%)	2007/08	Point layer	+	0.2100	DHS
*Biological susceptibility (BIO)*				*0.3728*	
Number of children under the age of 5^d^	2010	1 km	+	N/A	AfriPop:demography
Number of women of childbearing age	2010	1 km	+	0.0414	AfriPop:demography
Prevalence of stunting children under the age of 5	2010	5 arc-minutes	+	0.0843	FAO
Immunity	2010	1 km	-	0.1614	Malaria Atlas Project
HIV prevalence among 15-49 year olds (%)	2010	Polygon layer	+	0.0857	USAID
*Capacity to cope (C2C)*				*0.0857*	
Distance to closest hospital	2010	Point layer	+	0.0671	OSM, ESA GlobCover, SRTMv4
Number of dependents	2010	1 km	+	0.0186	AfriPop:demography

^a^Based on the outcomes of the literature survey, expert consultation and data availability; ^b^Refers to the spatial resolution of the original datasets (i.e., before the data was resampled to 10x10 km^2^ grids); ^c^Sign indicates if high indicator values increase (+) or decrease (-) vulnerability; ^d^This indicator was removed from the analysis to reduce existing multicollinearities in the data.

In their comprehensive reviews on risk and vulnerability to malaria, Bates et al. [17,18], Protopopoff et al. [27] and Sutherst [32] identify an entire set of (i) biological and disease-related (e.g., immunity, age, pregnancy, etc.), (ii) socioeconomic (e.g., socioeconomic status, poverty, nutritional status, education, etc.), as well as (iii) accessibility factors (e.g., access to health care, etc.), that impact people’s social vulnerability to malaria. In our paper, we consider these three groups of factors that determine malaria vulnerability in East Africa.

Many papers have been published on the mutual links between poverty and malaria [9,26,47,48]. There is strong evidence that poverty, or the lack of key capital assets, increases vulnerability to malaria through a number of factors. Bates et al. [17,18] highlight that there is a strong link between wealth and treatment-seeking behavior and access to malaria prevention services, such as ownership of nets, etc. We therefore used a dataset showing the spatial distribution of people living on less than two US$ per day, as provided by CGIAR CSI [49], as a proxy for poverty. Although several studies suggest that urbanization can result in a reduction of (i) places that could serve as potential *Anopheles* breeding sites, as well as (ii) transmission intensity [50], the initial process of rapid urbanization is often characterized by fast developing unplanned settlements and lacking basic infrastructure, and therefore often accompanied by increases in *Anopheles* larval habitats [27,50]. Lindsay and Martens [8] found that increased population density in the East African highlands resulted in an inevitable increase in human-vector contact, and thus increased the vulnerability to malaria. Thus, we have used increases in population densities from 1970 to 2010 as a proxy for urbanization. Civil and economic disturbances caused by violent conflicts or riots not only initiate the migration of people between different malaria transmission zones, and thus make them more susceptible (e.g. non-immune populations moving into endemic areas), but also impact people’s capacities to cope with, and recover from infection, as it hampers economic growth and destroys basic health and social service infrastructure [17,50]. We used time series from 1997 to 2009 derived from the Armed Conflict Location and Event Dataset (ACLED) to calculate a density layer (km^2^) of violent political conflict. As both the use of protection measures and treatment seeking behavior are influenced by perceptions, beliefs and knowledge about the disease [17,26,51-53], we integrated information on education levels derived from recent Demographic and Health (DHS) surveys into the analysis. For the geo-referenced DHS survey data gridded prevalence surfaces were created in *R* statistical software using the prevR package based on a workflow published by Lamarange et al. [54]. The use of mosquito nets, particularly by children under the age of five and pregnant women, is considered a key vulnerability indicator [9,17,26,27,29,47,48,55] as it has a tremendous impact on biting and infection rates. A variable from recent DHS surveys, indicating whether or not a child slept under a net the night before the survey, was integrated into the analysis to estimate the use of mosquito nets. Another key indicator is access to health care [9,18,27,47,48,56]. As the demand side (i.e., lack of available resources to cover costs, etc.) is partly covered by the poverty indicator, we have integrated distance to hospitals as a factor on the supply side, and as a key coping mechanism, into the analysis. The distance to health facilities is calculated as a cost distance depending on specific cost values for different land use/land cover (LULC) properties and considering topographical barriers (such as slope) using the path distance tool in ArcGIS. The tool calculates, for each grid cell, the least accumulative cost distance to the nearest source, while accounting for surface distance and horizontal (here: LULC) and vertical (here: elevation) cost factors. Thereby, LULC information was obtained from the GlobCover 2009 dataset, while the SRTMv4 dataset [57] was used to obtain elevation information. According to Bates et al. [17], evidence about the prevalence of malaria in male or female populations is still inconsistent. There is, however, evidence that gender has an influence on vulnerability in terms of different behavior, roles, expectations, and responsibilities, tending to make women more vulnerable to the disease [17]. We used gridded demographic population datasets provided by AfriPop:demography [58] to obtain information on the spatial distribution of the female population. Schneiderbauer [59] and Cutter et al. [60] indicate that a high dependency ratio (DR) in a given area can impact people’s susceptibility in several ways. Although their findings primarily relate to vulnerability to natural hazards in a DRR framework, a high DR also impacts malaria vulnerability by imposing a higher economic burden on the working population, thus leaving fewer resources for coping with the disease in case of infection or severe illness. We have therefore integrated DR into the analysis, as measured by the number of dependents (below 15, and above 65 years) as a percentage of the working-age group between 15 and 64 years of age, based on the population datasets provided by AfriPop.

We also integrated distance to road networks, using data provided by OpenStreetMap, and travel time to local markets into the analysis. For the latter we used a gridded accessibility surface provided by the World Bank and JRC (http://bioval.jrc.ec.europa.eu/products/gam/index.htm) as a proxy [61]. Accessibility to road networks and transport is often perceived as a relevant development indicator covering generic access to a variety of services [62,63]. As local markets and urban centers are important central places, which links to the central place theory of Christaller [64], these have been included to reflect the availability of alternative livelihoods as well as the access to sales market [65].

Several studies have shown that, due to lowered immunity and impaired efficacy of antimalarial drugs during pregnancy, both pregnant women and children under five, are particularly susceptible [17,27,47,66,67]. As up-to-date data on current pregnancy status was not available for the entire study area, the number of women of childbearing age (15-49 years), as provided by AfriPop, was used as a proxy for biological susceptibility (BIO). Aside from pregnant women and young children, it is particularly the communities in the highlands that are vulnerable. Their immunity is lower compared to their counterparts in the lowlands [9]. As immunity generally develops with increasing malaria transmission, we used the age-standardized *P. falciparum* parasite rate, which describes the estimated proportion of 2-10 year olds in the general population that are infected with *P. falciparum* at any one time, averaged over the 12 months of 2010 [24] as a proxy for immunity. In the absence of data on immunity status of the population it was considered a reasonable proxy for biological susceptibility. The current scientific debate on the relationship between malnutrition and susceptibility to malaria is still blurred. While some studies suggest that poor nutritional status increases susceptibility [17,68], others found that nutritional stress might even be protective against malaria [69,70]. Ultimately, there are also studies that revealed no clear link between nutritional status and susceptibility [71]. However, evidence is accumulating that poor nutritional status has an impact on people’s susceptibility [27]. Thus, in the absence of reliable data for the entire region, the authors have used the number of stunting children under the age of five, as provided by the Food and Agriculture Organization (FAO), as a proxy for poor nutritional status in children. According to Bates et al. [17] there is increasing evidence that HIV co-infection leaves people more vulnerable to malaria. We therefore included HIV-prevalence among 15-49 year olds in the analysis; as acquired from UNAIDS. As HIV-prevalence was reported on district level, we disaggregated this information using population information provided by AfriPop.

### Modeling homogeneous regions of social vulnerability

Based on a concept and methodology for modeling multi-dimensional, latent spatial phenomena, we modeled relative levels of social malaria vulnerability on a regional scale. Our approach builds on the concept of geons which was introduced by Lang et al. [72]. Recently, Lang et al. [73] defined geons as spatial objects, which are homogenous in terms of varying spatial phenomena under the influence of policy intervention and are generated by scale-specific spatial regionalization of a complex, multidimensional geographical reality incorporating expert knowledge. In this paper we follow the concept of *integrated geons*[73], which addresses abstract, yet policy-relevant phenomena such as societal vulnerability to hazards.

The methodology to delineate integrated geons was initially developed by Kienberger et al. [74] and has been successfully applied to model vulnerability to floods at different spatial scales [75], as well as to identify hotspots of cumulative climate change impact in Western Africa [76]. This paper presents an expanded methodology to represent integrated geons incorporating methods for indicator preprocessing and sensitivity analysis. Integrated geons, i.e. homogenous regions of social vulnerability to malaria, are delineated using a workflow that comprises five major stages (Figure 3).

First, the conceptual framework is defined (see Figure 2) to provide guidance on how to best represent and operationalize the phenomenon of concern. This step also includes the identification and first selection of possible indicators and datasets relevant for the specific VBD that is addressed. These indicators should fulfill three specific criteria to be considered suitable: salience, credibility and legitimacy [77]. Additionally, it is important that data are suitable to represent the indicators in a spatially-disaggregated manner.

Within the second stage, different pre-processing routines are carried out to prepare datasets for modeling, and to test the statistical soundness of the indicator framework. This includes creating gridded surfaces (here: 10 × 10 km^2^), cropping them to the extent of the modeling region, as well as the identification and treatment of outliers, missing data and multicollinearities in the data. To create the 10 × 10 km^2^ grids, some of the indicators, including number of women, population change, travel time to closest urban center, etc., were resampled from smaller cell sizes, while HIV prevalence – which was reported on sub-national administrative units – was disaggregated using a gridded population dataset acquired from WorldPop. Outliers were identified using box plots, and treated by applying a 3 × 3 customized low pass filter which reduces extreme values by replacing them with the mean values of the eight neighboring pixel values. Outliers were treated for the following datasets: children under the age of 5, women of childbearing age, stunting children under the age of 5, number of HIV-infected persons. Multicollinearities were assessed using the Pearson correlation coefficient *r*, and considering the variance inflation factor (VIF); with *r* > 0.9 or VIF > 5 indicating a multicollinearity problem [78]. Based on these statistics the variable children under the age of 5 was removed from the analysis, as it was highly collinear with stunting children under the age of 5 (see Table 1). As a final step in stage 2, all indicators were normalized to an 8-bit interval [0, 255] using linear min-max normalization (Equation 1).

(1) vi'=vi−vminvmax−vmin∗255

where *v*_*i*_ refers to the raw pixel value, and *v*_*min*_ and *v*_*max*_ represent the minimum and maximum values of the raw pixel value respectively. During normalization, the indicators were adjusted for their sign, which indicates whether the indicator contributes positively (+) or negatively (-) to vulnerability (Table 1). This was done by multiplying the respective indicators by minus one, and then adding their minimum value. This results in datasets where high values increase vulnerability and low values decrease vulnerability.

A set of integrated geons was delineated in the third stage. This was achieved by regionalizing the weighted indicators in an *n*-dimensional indicator space using the multi-resolution segmentation algorithm [79] implemented in the TRIMBLE eCognition Developer software environment. To evaluate the relevance of each indicator for malaria vulnerability in the study area a weight for each indicator was obtained from an expert-based weighting exercise. In total, seven regional domain experts of varying backgrounds (such as epidemiologists, health ministries, climate, and health specialists) with long-term malaria expertise in the region participated in the survey. Making use of an online survey the experts were asked to allocate 100 points to the final set of vulnerability indicators. By taking the mean value of the seven expert ratings, and standardizing them to sum up to one, we came up with a weight for each of the 14 indicators, as listed in Table 1. The size of the regions depends on the parameterization of the segmentation algorithm, which can be adjusted by the user. We used the ‘Estimation of Scale Parameter’ (ESP2) tool [80] to identify the statistically most suitable scale parameterization of the algorithm. Following the conceptual framework (Figure 2) and its sequential relationship between the four vulnerability domains we delineated regions of social vulnerability using a step-wise approach: A first set of integrated geons was delineated based on the six weighted ‘generic susceptibility’ indicators. Based on these units, we used the two weighted ‘lack of capacity to anticipate’ indicators to refine the regions. Then, the four weighted ‘biological susceptibility’ and the two weighted ‘lack of capacity to cope’ indicators were sequentially integrated into the analysis. The resulting fine-scaled units or geons were ‘merged’ considering all indicators; again applying the multi-resolution segmentation algorithm. For each regionalization step, the scale parameter – which determines the size of the unit based on homogeneity criteria – was identified using the ESP2 tool [80]. Following this step-wise approach we are able to represent relationships between the different vulnerability domains; as indicated in the conceptual vulnerability framework.

A final vulnerability index value is calculated for each geon using the weighted vector magnitude according to the following equation [73,74]:

(2) $$I_{\mathrm{VU}}=\;\sqrt{w_{\mathrm{sus}}\mathrm{SU}S^{2}+\;w_{c2a}C2A^{2}+\;w_{\mathrm{bio}}\mathrm{BI}O_{\ldots }^{2}+\;w_{c2c}C2C^{2}\;}$$

where *I*_*VU*_ refers to the social vulnerability index for each integrated geon, *SUS, C2A, BIO and C2C* to the indices for the four vulnerability domains, and *w* to the aggregated weights for each domain. The index values for each of the four domains are also calculated using the weighted vector magnitude (Equation 3):

(3) $$I_{\mathrm{DOM}}=\;\sqrt{w_{1}v{'}_{\mathrm{DO}M_{1}}^{2}+\;w_{2}v_{\mathrm{DO}M_{2}}^{'2}+\;w_{\ldots }v_{\ldots }^{'2}+\;w_{n}v_{\mathrm{DO}M_{n}}^{'2}\;}$$

where *I*_*DOM*_ refers to the index for each of the four vulnerability domains (SUS, C2A. BIO and C2C), *v*_*DOMi-n*_ to the normalized indicators identified for each domain (Table 1) and *w*_*i-n*_ to the expert-based indicator weights.

To ease the interpretation of the results, the resulting vulnerability index values for each unit were normalized to the zero to one interval [0,1], where zero represents no, and one very high vulnerability to malaria on a relative scale within the case study region.

To assess the robustness of the modeling approach in regard to the choice of indicators we performed a local sensitivity analysis. Therefore, following an approach described in Lung et al. [81] we calculated a set of alternative vulnerability indices by discarding one indicator at a time while keeping all other settings (normalization, weighting, aggregation) equal. The outputs of this approach are presented in the results section.

In a final step, the index values are mapped and visualized using a blue (low value) to red (high value) color scheme to avoid difficulties for the color blind. We refrained from a classification of the index values into categories and visualized each unit based on its index value using a continuous color scheme instead.

## Results

### Social vulnerability to malaria

**Figure 4:**
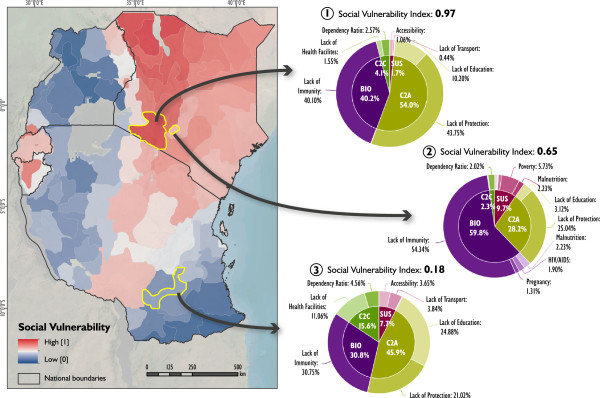
**Social vulnerability to malaria in eastern Africa.** Figure 4 shows current levels of social vulnerability to malaria in East Africa. The pie-charts show the varying contribution of the single vulnerability indicators for different selected geons. Such pie-charts can be visualized for each geon, thus guiding the identification of targeted intervention options.

Figure 4 shows the spatial distribution of social vulnerability to malaria for the EAC region. In the map, areas of high vulnerability are displayed in red (max value = 1), while areas of low vulnerability (min value = 0) are displayed in blue using the continuous classification scheme. Regions of very high vulnerability are found in the northeastern part of the study area, particularly in the areas surrounding Lake Turkana, Kenya, at the Kenyan-Ugandan and Kenyan-Tanzanian border, as well as in the central part of Burundi. Medium to high levels are found in Rwanda with very high levels in Kigali, as well as in the northeastern and southwestern part of Tanzania. The pie charts for three selected vulnerability regions in Figure 4 indicate the relative share and contribution of the underlying vulnerability indicators to the overall vulnerability index; thus enabling an evaluation of different characteristics for each integrated geon.Although high levels of vulnerability in areas that are currently malaria free, or only affected by epidemic outbreaks, such as the East African highlands (see Figure 1), seem surprising at first glance, this is primarily a result of the lowered immunity of the populations in these regions. As vulnerability is seen as of two key components of risk, it represents the societal predisposition which is independent from the current distribution of infected vectors. The decomposition of risk into its underlying components of hazard (i.e., probability of an infective bite) and vulnerability is useful, as it helps to identify potential future areas at-risk, as well as targeting relevant societal drivers.

**Figure 5:**
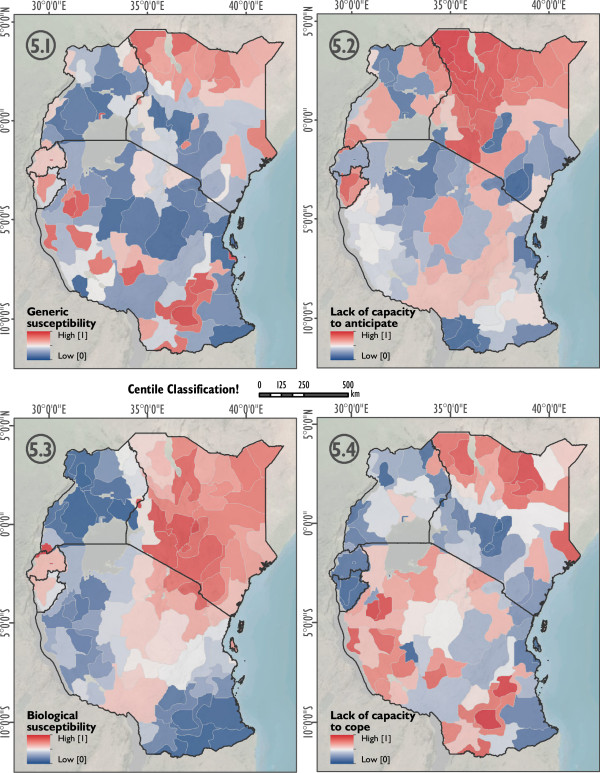
**Domains of social vulnerability to malaria in east Africa.** Figure 5 shows the domains of social vulnerability to malaria based on centile classification. Generic susceptibility (5.1), lack of capacity to anticipate (5.2), biological susceptibility (5.3), and the lack of capacity to cope (5.4).

As an additional output, Figure 5 (5.1 to 5.4) displays the spatial heterogeneity of generic susceptibility (Figure 5.1), the lack of capacity to anticipate the disease (Figure 5.2), biological susceptibilities (Figure 5.3), and the lack of capacity to cope with the disease (Figure 5.4) in the study area. While generic susceptibility is rather low in the region, biological susceptibility is generally high, especially in areas where *Pf endemicity* is low (see Figure 1); due to a lack of immunity.

### Influence of input indicators on the composite vulnerability index

**Figure 6:**
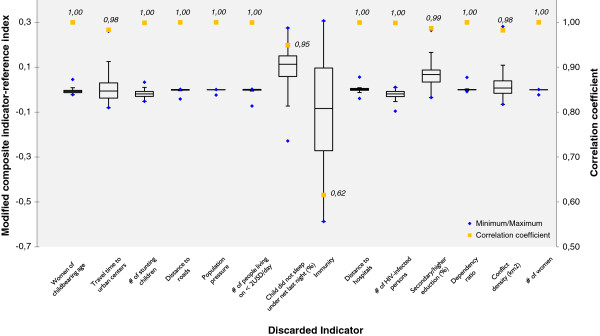
Box plots showing the influence of the single indicators on the composite vulnerability index.

As outlined above, the modeling of homogeneous vulnerability units comprises several stages where the analyst is confronted with choices between different plausible alternatives that impact the modeling outcome [82]; in our case the size and shape of the integrated geons as well as the vulnerability index. It is therefore important to analyze the impact of these choices by assessing the sensitivity of the modeling approach, as well as related uncertainties [82,83]. Sensitivity analysis evaluates the contribution of individual sources of uncertainty to the output variance [84,85]. In contrast to global sensitivity analysis, which enables a simultaneous assessment of multiple construction stages, local sensitivity analysis targets one construction stage at a time, while all other stages are held constant [82]. As no framework (so far) exists for assessing the global sensitivity and uncertainty for geons [73], we assessed the influence of the input vulnerability indicators on the vulnerability index by means of a local sensitivity analysis. This was achieved by discarding one of the indicators at a time, while keeping all other settings (normalization, weighting, regionalization, and aggregation) equal [81], and resulted in a series of alternative vulnerability indices. For each geon the alternative index was compared with the reference vulnerability index (i.e., the index based on all indicators). The results are displayed in the box plots in Figure 6, which, for each of the alternative vulnerability indices (x-axis), show the interquartile range (IQR), the minimum and maximum values as well as the correlation (*r*) with the reference index (y-axis). The higher the IQR, the higher the influence of the respective indicator on the vulnerability index [81].

The box plots and the correlation coefficients (Pearson’s *r*) displayed in Figure 6 clearly show that children not sleeping under a net (*r* = 0.95), travel time to the closest urban center (*r* = 0.98), education (*r* = 0.99) and conflict density (*r* = 0.98) have a minor impact, while immunity (*r* = 0.62) has a marked impact on the vulnerability index. With the exception of the indicator ‘immunity’, which has an excessive influence on the composite vulnerability index, the strong correlation between the modified vulnerability indices and the reference vulnerability index (*r* never smaller than 0.95) emphasizes the robustness of the vulnerability index in regard to the final choice of input datasets; again with the exception of the indicator related to immunity which has a marked impact on the composite vulnerability index.

## Discussion

As shown in Figures 4 and 5, vulnerability – and its decomposed domains – varies significantly in space. This is a result of a spatial variation of the underlying vulnerability indicators. Figure 4 allows the identification of social vulnerability hot spots for malaria on a relative scale for East Africa. The results are useful for decisions regarding the entire eastern African scale level, as it supports the rough identification of intervention areas. The answer to the question “what needs to be done where?” can be derived by exploring the relative share of contributing vulnerability indicators as depicted in the pie-charts in Figure 4. For instance, region 2 – representing the urban region of Nairobi – has a stronger contribution of biological susceptibility than the neighboring region 1, or region 3. The pie-charts in Figure 4 also show that a lack of immunity is a major contribution to malaria vulnerability in the study area, which is also a result of the relatively high weight that was assigned to this indicator by the experts. However, at the same time, it also becomes evident that a lack of immunity is only one of several important factors contributing to malaria vulnerability in the study area, which is also reflected by the weights that were assigned to the single indicators by the experts (see Table 1). Aside from immunity, other relevant indicators include the lack in use of protection measures (i.e., the lack in use of bed nets), poverty, distance to hospitals, and lack of education, amongst others. This has important policy making implications, since interventions that aim at reducing the burden of the disease should not only be spatially targeted, but also take into account the relevance of each of these factors for malaria vulnerability for the respective regions. Furthermore, it is also interesting that for instance Nairobi (Figure 4, region 1) is delineated as a homogenous region. Although the presented approach does not include any information on administrative boundaries, it well reflects a homogeneous urban region which differs from its surrounding area in terms of its socioeconomic and demographic characteristics.Figure 5 presents the different indices for the four domains of vulnerability. Care has to be taken with the interpretation, as the generic susceptibility (SUS) domain has a significantly higher value in Kigali in regard to female population. To allow a comparison of the four domains the values have been classified with centile classifications.

The benefit of the geon approach as presented in this paper is that it delineates homogenous regions which are independent of *a-priori* geographies [86], such as administrative boundaries, and therefore facilitates a place-specific identification of possible interventions. As administrative boundaries are artificially drawn and may change over time, they can have a direct influence on the aggregated index value. For further details we refer to the Modifiable Areal Unit Problem (MAUP) as discussed by Openshaw [87]. A discussion on MAUP for geons is provided by Lang et al. [73]. Additionally, the size and shape of administrative boundaries varies significantly within the study area (e.g. district boundaries in Rwanda vs. district boundaries in Tanzania), and are not an objective measure or suitable for a relative, spatial evaluation of vulnerability across the region. We do, however, not neglect the importance of administrative boundaries as reporting units for the implementation of malaria policies and interventions [30].

From the methodological point of view this paper advances beyond the initial workflow discussed by Kienberger et al. [74] through the application of pre-processing methods and an advanced delineation of the vulnerability regions. Now, it includes methods for (i) pre-processing of indicators and statistical testing of the soundness of the indicator framework (based on OECD [78] and Hagenlocher et al. [46]), (ii) the identification of a statistical valid scale parameter, as well as for (iii) local sensitivity analysis. In the absence of causal models that evaluate the contribution of the indicators considered for social vulnerability in the study area, indicator weights were identified based on expert opinions. An alternative modeling exercise could be based on statistical weighting procedures, e.g. using weights based on principal component analysis (PCA) or regression analysis. In a previous study, we compared both statistical and expert-based weighting schemes, evaluated their impact on a vulnerability index in Cali, Colombia, and found that both modeling approaches revealed similar outputs, both globally and spatially [46].

Additionally, moving from a local sensitivity analysis approach towards a global sensitivity analysis, which considers the influence of indicators, normalization, weighting and aggregation, will be part of future research. This is particularly challenging when using geons, as not only the vulnerability index for each geon changes when altering input parameters, but also the geometry of the geons might change. To overcome this challenge we are currently developing metrics to quantify these impacts, and ultimately provide information on the stability of the delineated geons. Future research will also consider spatially explicit approaches for indicator pre-processing.

Critical for such assessments is the quality and availability of input data. An increasing number of disaggregated and spatially explicit data is publically available. However, due to its multi-source characteristic, data quality and accuracy varies between regions and datasets. As the data used for this study includes uncertainties, the results of such modeling exercises as presented here are mainly for (i) indicative purposes, and (ii) valid only for a regional scale level. Information on vulnerability not yet covered by the proposed set of indicators includes data on the quality of health services or interventions such as indoor residual spraying (IRS). Once such data are available for the entire study area, this could additionally reduce existing uncertainties in the spatial assessment of social vulnerability.

To achieve the ultimate aim of spatially explicit risk assessment, the outcomes of the presented vulnerability analysis should be combined with information on the probability of an infective malaria bite (e.g. represented through the EIR). This would allow a validation of the results based on field measurements of malaria prevalence, using for example the results of rapid diagnostic tests (RDTs). As shown in Figure 4, the presented approach provides the opportunity to integrate the modeling outcomes as well as the underlying indicator framework into an interactive web-environment [88], which can serve as a simple spatial decision support tool.

## Conclusions

An expert-based, spatially explicit approach was utilized for modeling and visualizing relative levels of prevailing social vulnerability to malaria in the Eastern African Community (EAC) region. Taking into account a set of socioeconomic, demographic, access and biological/disease-related indicators, vulnerability to malaria was modeled independent of the current spatial distribution of the disease. In the context of a changing environment it is of utmost importance not only to target areas that are currently malaria endemic, but also to focus on areas that might be affected by the disease in the near future due to a changing climate and its societal drivers. A holistic risk and vulnerability framework was developed and used as a heuristic guidance tool for the identification and development of a sound indicator framework, thus enabling a reproducibility or transferability of results. The results of our research provide relevant information for policy makers to identify place-specific interventions that decrease people’s susceptibility to the disease and help to strengthen their resilience. Combined with information on disease prevalence, this is one important step towards a more integrative and systemic view of malaria risk.

## Competing interests

The authors declare that they have no competing interests.

## Authors’ contributions

SK and MH developed the study design and were responsible for the conceptualization of the study. MH and SK did the literature review and data collection. MH was responsible for data pre-processing, the analysis, and the design and implementation of the expert survey, where SK provided supervision. Both interpreted the results and wrote the paper. The authors read and approved the final version of the manuscript.
